# Drug repositioning in non-small cell lung cancer (NSCLC) using gene co-expression and drug–gene interaction networks analysis

**DOI:** 10.1038/s41598-022-13719-8

**Published:** 2022-06-08

**Authors:** Habib MotieGhader, Parinaz Tabrizi-Nezhadi, Mahshid Deldar Abad Paskeh, Behzad Baradaran, Ahad Mokhtarzadeh, Mehrdad Hashemi, Hossein Lanjanian, Seyed Mehdi Jazayeri, Masoud Maleki, Ehsan Khodadadi, Sajjad Nematzadeh, Farzad Kiani, Mazaher Maghsoudloo, Ali Masoudi-Nejad

**Affiliations:** 1 Department of Biology, Tabriz Branch, Islamic Azad University, Tabriz, Iran; 2 Department of Health Ecosystem, Medical Faculty, Nisantasi University, Istanbul, Turkey; 3 Farhikhtegan Medical Convergence Sciences Research Center, Farhikhtegan Hospital Tehran Medical Sciences, Islamic Azad University, Tehran, Iran; 4 Immunology Research Center, Tabriz University of Medical Sciences, Tabriz, Iran; 5 Department of Genetics, Faculty of Advanced Science and Technology, Tehran Medical Sciences, Islamic Azad University, Tehran, Iran; 6 Molecular Biology and Genetics Department, Engineering and Natural Science Faculty, Istinye University, Istanbul, Turkey; 7 Departamento de Biología, Universidad Nacional de Colombia, Bogotá, Colombia; 8 Department of Agronomy and Plant Breeding, Tabriz Branch, Islamic Azad University, Tabriz, Iran; 9 Department of Computer Engineering, Faculty of Engineering and Architecture, Nisantasi University, Istanbul, Turkey; 10 Software Engineering Department, Faculty of Engineering and Natural Sciences, Istinye University, Istanbul, Turkey; 11 Laboratory of Systems Biology and Bioinformatics (LBB), Institute of Biochemistry and Biophysics, University of Tehran, Tehran, Iran

**Keywords:** Non-small-cell lung cancer, Drug discovery, Computational models

## Abstract

Lung cancer is the most common cancer in men and women. This cancer is divided into two main types, namely non-small cell lung cancer (NSCLC) and small cell lung cancer (SCLC). Around 85 to 90 percent of lung cancers are NSCLC. Repositioning potent candidate drugs in NSCLC treatment is one of the important topics in cancer studies. Drug repositioning (DR) or drug repurposing is a method for identifying new therapeutic uses of existing drugs. The current study applies a computational drug repositioning method to identify candidate drugs to treat NSCLC patients. To this end, at first, the transcriptomics profile of NSCLC and healthy (control) samples was obtained from the GEO database with the accession number GSE21933. Then, the gene co-expression network was reconstructed for NSCLC samples using the WGCNA, and two significant purple and magenta gene modules were extracted. Next, a list of transcription factor genes that regulate *purple* and *magenta* modules' genes was extracted from the TRRUST V2.0 online database, and the TF–TG (transcription factors–target genes) network was drawn. Afterward, a list of drugs targeting TF–TG genes was obtained from the DGIdb V4.0 database, and two drug–gene interaction networks, including drug-TG and drug-TF, were drawn. After analyzing gene co-expression TF–TG, and drug–gene interaction networks, 16 drugs were selected as potent candidates for NSCLC treatment. Out of 16 selected drugs, nine drugs, namely *Methotrexate, Olanzapine, Haloperidol, Fluorouracil, Nifedipine, Paclitaxel, Verapamil, Dexamethasone, and Docetaxel,* were chosen from the drug-TG sub-network. In addition, nine drugs, including *Cisplatin, Daunorubicin, Dexamethasone, Methotrexate, Hydrocortisone, Doxorubicin, Azacitidine, Vorinostat, and Doxorubicin Hydrochloride,* were selected from the drug-TF sub-network. *Methotrexate* and *Dexamethasone* are common in drug-TG and drug-TF sub-networks. In conclusion, this study proposed 16 drugs as potent candidates for NSCLC treatment through analyzing gene co-expression, TF–TG, and drug–gene interaction networks.

## Introduction

Lung cancer is one of the leading cancer death causes^1^ worldwide. This type of cancer occurs when a cancerous tumor grows inside the lungs. Lung cancer contains two main types: non-small cell lung cancer (NSCLC) and small cell lung cancer (SCLC). NSCLC is the most common lung cancer^2^. Histopathological grading has identified about 85% to 90% of lung cancers as NSCLC and 15% to 20% as SCLC^3^. This cancer includes three different types of Adenocarcinoma, Squamous cell carcinoma, and large cell carcinoma.

Different studies based on computational approaches and network analysis have been undertaken to find biomarker genes for early NSCLC detection. Moreover, scientists have evaluated and discussed the effect of current drugs on this cancer. Based on a co-expression network analysis, Ling Kui et al.4 proposed several important genes as biomarkers for NSCLC treatment. Xiujuan Gao et al.^4^ applied the gene expression profile of NSCLC samples and, based on a systems biology approach, reported Estrogen receptors (ERs) as promoters of NSCLC progression. In another study, Mei Zhao et al.^5^ introduced five genes, including *FGF2*, *GOLM1*, *GPC3*, *IL6,* and *SPP1,* which deregulated in NSCLC tissues. They introduced these 5 genes for NSCLC prognosis in patients. A computational approach based on protein–protein interaction (PPI) network analysis was used in a similar study, and Stratifin had an important role in NSCLC^6^ development. Furthermore, Yun-Qiang Zhang et al.^7^ proposed *HIST1H2BH* and *PLK1* as prognostic biomarkers for NSCLC patients.

Drug repositioning (DR) is utilized as a time- and cost-effective method to discover new drugs^8–12^. Drug repositioning is also referred to as drug repurposing, drug therapeutic, drug recycling, and drug reprofiling^13,14^. There are usually three kinds of methods for drug repurposing, including experimental biological methods, computational methods, and mixed methods^10,15,16^. Computational methods can be referred to as molecular docking, network mapping, signature matching, genetic association, and retrospective clinical analysis^13,17,18^. In the current study, a computational drug repositioning method is applied to identify candidate drugs to treat NSCLC.

Lately, various studies based on the network approach for drug repurposing have been carried out. Network-based strategy is one of the important computational methods in drug repurposing^19,20^. SAveRUNNER^21^ is a network-based algorithm in this field. This algorithm predicts drug-disease relations based on a similarity measure. This method was provided as an R programing language package^22^. Xing Li and colleagues ^23^ proposed a network-based approach to discover lncRNA biomarkers in human lung adenocarcinoma. Furthermore, a computational approach for drug repurposing based on the system biology approach was proposed by Azam Peyvandipour and collages ^24^ in 2018. In another study, Wei-Feng Guo et al.^25^ proposed a network controllability-based algorithm called combinatorial drug identification algorithm (CPGD). Besides, Albert Li and collages^26^ proposed a network-based method, namely LncTx, to repurpose drugs in lung cancer. In a recent study by Zahra and her colleagues^27^, they proposed a novel network-based method to discover candidate drugs for bladder cancer.

Anisha et al. ^28^ presented an overview of drug repositioning for anti-cancer applications, and they proposed a novel drug repurposing technique to target the MAPK signaling pathway in NSCLC. In a similar study, Muthu Kumar and colleagues^29^ introduced another drug repurposing method for NSCLC, and they hypothesized that Nebivolol is an excellent candidate for inhibiting MEK in NSCLC patients. In another study, Joelle C. Boulos and colleagues^30^ repurposed ALK Inhibitor Crizotinib for NSCLC, Acute Leukemia, and Multiple Myeloma Cells. Compared to the mentioned methods, this study applies a novel computational model based on gene co-expression and TF–TG interaction networks. Moreover, two drug–gene interaction networks, including drug-TF and drug-TG, were studied that have not been studied in previous studies.

Gene co-expression network analysis is one of the important network-based approaches in systems biology^11,31,32^. Different studies based on gene co-expression network analysis were done on different transcriptomic datasets. In this study, a gene co-expression network analysis was applied on the NSCLC transcriptomics dataset to repurpose some potent candidate drugs for NSCLC. Xue-Tao Li ^33^ and colleagues applied gene co-expression modules analysis in order to predict non-small cell lung cancer survivals. In a similar project, Guanghui Wang et al.^34^ applied gene co-expression modules analysis on NSCLC metastases.

Weighted gene co-expression network analysis (WGCNA^35^) is a bioinformatics and systems biology tool that is employed to construct and analyze co-expression networks. This tool is an R programming language package and contains different functions for network construction, visualization, data simulation and gene selection and can be applied for detecting modules (clusters) of highly correlated genes^36^. In the present study, WGCNA was utilized to reconstruct and analyze the gene co-expression network for NSCLC transcriptomic dataset. Xuting Xu and colleagues ^37^ applied WGCNA to identify hub genes as biomarkers in lung cancer and introduced *CCNB1*, *CCNE2*, *MCM7*, and *PCNA* as hub biomarker genes. In a similar study, Binglin Chen et al.^38^ applied WGCNA on the NSCLC transcriptomics dataset and identified four hub genes (*AURKB, CDC20, TPX2 , and KIF2C*) as NSCLC prognostic biomarkers based on co-expression network analysis. Moreover, WGCNA was utilized to discover prognostic markers in lung cancer by Bo Ling colleague ^39^.

The current study aimed to discover potent candidate drugs for NSCLC treatment by analyzing gene co-expression, TF–TG, and drug–gene interaction networks. To this end, at first, a gene co-expression network was reconstructed based on the WGCNA for the NSCLC transcriptome dataset. Then, two significant gene modules, named *purple* and *magenta,* were discovered from the reconstructed gene co-expression network. Next, a list of transcription factor (TF) genes regulating *purple* and *magenta* modules' genes was gathered from the TRRUST V2.0^40^ online database. Afterward, a TF-gene interaction network was reconstructed for the gathered TFs and their target genes. This network is named the TF–TG network. Simultaneously, Gene Ontology (GO) and pathway enrichment analysis were done using the David bioinformatics Resources 6.8^41^ for *purple* and *magenta* modules' genes, and the results were reported. Subsequently, in order to identify the existing drugs targeting TF–TG network genes, the DGIdb V4.0^42^ online database was utilized. After obtaining a list of drugs targeting the TF–TG genes, we reconstructed two drug–gene interaction networks, including drug-TF and drug-TG. Consequently, for each of the drug-TF and Drug-TG networks, nine high-degree drugs (hub drugs) were selected and reported as potent candidate drugs. METHOTREXATE is a hub node in the drug-TG interaction network and regulates 6 genes of the *purple* and *magenta* modules. The highest degree drug node in the drug-TF interaction network is *CISPLATIN,* which regulates 11 TF genes.

**Figure 1:**
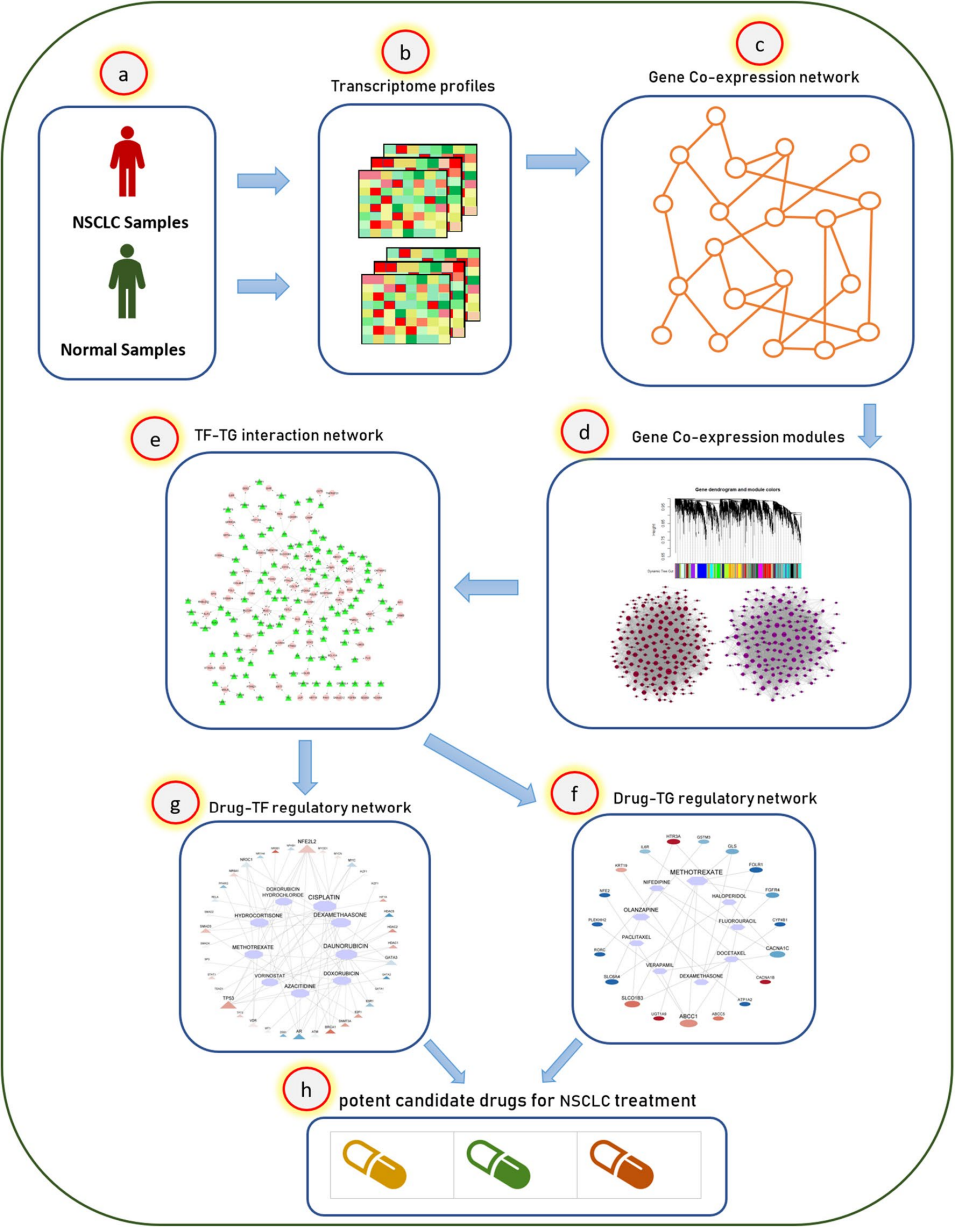
The workflow diagram of the proposed method. This study applies a gene co-expression network and a drug–gene regulatory network analysis to reposition candidate drugs for NSCLC treatment. **(a,b)** At first, a transcriptome profile for normal and NSCLC samples was downloaded from the GEO database with the accession number GSE21933. **(c,d)** Then, a gene co-expression network was reconstructed for the differentially expressed genes (p_value < 0.01) of normal and NSCLC groups using the WGCNA package in the R programming environment, and two significant gene modules (*purple* and *magenta*) were extracted from the NSCLC co-expression network. **(e)** Next, a list of transcription factor genes, which regulate *purple* and *magenta* modules' genes, were obtained from the Trrust V2.0 ^40^ online database. **(f,g)** Subsequently, two drug–gene interaction networks, named drug-TG (target gene) and drug-TF (transcription factor gene), were drawn using the DGIdb V4.0^42^ online database. **(e)** Finally, 18 candidate drugs are proposed for NSCLC treatment.

In summary, the current study consists of the following three main steps: (1) Gene co-expression network reconstruction, (2) TF_TG interaction network analysis, and (c) Drug- Target interaction network analysis. Compared to the other studies, steps (2) and (3) are novel in our project and have not been applied for drug repurposing for NSCLC before. Moreover, the current study analyses interactions between drugs and both of TFs and non-TF genes (Drug-TF and Drug-TG), which have not been studied before for NSCLC treatment. Figure 1 shows the workflow diagram of the proposed approach.

## Result

### Module analysis

For 4218 differentially expressed genes between normal and NSCLC, the gene co-expression network was reconstructed for NSCLC transcriptomics data using the WGCNA. Accordingly, 21 gene modules were discovered from this network (Supplementary Fig. C). The *darkorange* module is the smallest module with 47 genes, while the largest module is *blue* with 326 genes. The *grey* module shows genes that are not assigned to any other detected modules. This module is not considered for further analysis.

### Comparing the modules between NSCLC and normal groups

**Figure 2:**
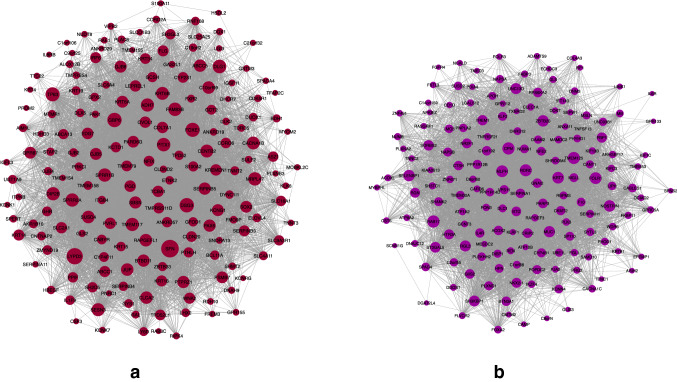
*Magenta* **(a)** and *Purple* **(b)** modules. The circle nodes represent genes (this figure was drawn in the Cytoscape^43^ v.3.8.2 software).

Those modules that have changed significantly between NSCLC and normal groups could deregulate some biological processes and cause disease. Therefore, no-preserve modules between NSCLC and normal groups may cause the NSCLC. As described in the method section, the modules with ${{\varvec{Z}}}_{{\varvec{s}}{\varvec{u}}{\varvec{m}}{\varvec{m}}{\varvec{a}}{\varvec{r}}{\varvec{y}}}<2$ are considered as no preservation modules for additional analysis. In this regard, the *purple* and *magenta* modules have ${{\varvec{Z}}}_{{\varvec{s}}{\varvec{u}}{\varvec{m}}{\varvec{m}}{\varvec{a}}{\varvec{r}}{\varvec{y}}}=0.93$ and ${{\varvec{Z}}}_{{\varvec{s}}{\varvec{u}}{\varvec{m}}{\varvec{m}}{\varvec{a}}{\varvec{r}}{\varvec{y}}}=1.3$, respectively and are considered as no preservation modules between NSCLC and normal groups (see Fig. 2). These modules can represent cancer progression from normal to NSCLC stage. Table 1 shows all extracted modules along with their ${{\varvec{Z}}}_{{\varvec{s}}{\varvec{u}}{\varvec{m}}{\varvec{m}}{\varvec{a}}{\varvec{r}}{\varvec{y}}}$.

**Table 1:** The ${{\varvec{Z}}}_{{\varvec{s}}{\varvec{u}}{\varvec{m}}{\varvec{m}}{\varvec{a}}{\varvec{r}}{\varvec{y}}}$ of NSCLC co-expression modules compared to the normal gene expression data.

Module name	Size	${Z}_{summary}$
Purple	167	0.93
Magenta	183	1.3
Orange	52	2.2
Darkgreen	81	2.7
Red	222	3.5
Grey60	101	4
Midnightblue	112	4.3
Greenyellow	160	4.6
Lightgreen	92	4.6
Cyan	133	5.5
Darkred	84	5.5
Lightcyan	108	5.8
Lightyellow	90	5.8
Darkturquoise	70	6.7
Brown	317	6.9
Blue	326	7.4
Royalblue	87	8.1
Darkorange	47	10
Salmon	140	14
Gold		17
Black	192	27
Grey	42	0.46

### Enrichment analysis of the gene modules

In order to study the biological functions of the genes in *purple* and *magenta* modules, functional enrichment analysis was performed using the DAVID^5^ (Database for Annotation Visualization and Integrated Discovery) database. Gene Ontology (GO) enrichment analysis shows that the genes of *purple and magenta* modules* are* enriched in 55 and 72 significant (p_value < 0.05) terms, respectively. The results show that the genes in the *purple* module are significantly enriched in some biological processes related to respiration and lung including: *lung epithelial cell differentiation (p_value* < *0.001), lung cell differentiation(p_value* < *0.001), lung epithelium development (p_value* < *0.004), respiratory system development (p_value* < *0.006),* and *lung development (p_value* < *0.01).* As well as, the results show that the genes in the *magenta* module are not significantly enriched in biological processes related to respiration and lung. Therefore, the *purple* module genes are closer to NSCLC than the *magenta* module. More details for the GO results are reported in supplementary file S2.

Moreover, to investigate biological pathways related to the purple and magenta modules, the pathway enrichment analysis was done based on the REACTOME^44^ database. The results revealed that the *purple* module is significantly enriched in the regulation of the *insulin secretion* pathway. In addition, the *magenta* module is significantly enriched in five biological pathways, including *Gap junction assembly, TP53 Regulates Metabolic Genes, Tandem of pore domain in a weak inwardly rectifying K* + *channels (TWIK), Tight junction interactions,* and* Synthesis of 12-eicosatetraenoic acid derivatives* (see supplementary file S2).

### TF–TG regulatory network

**Figure 3:**
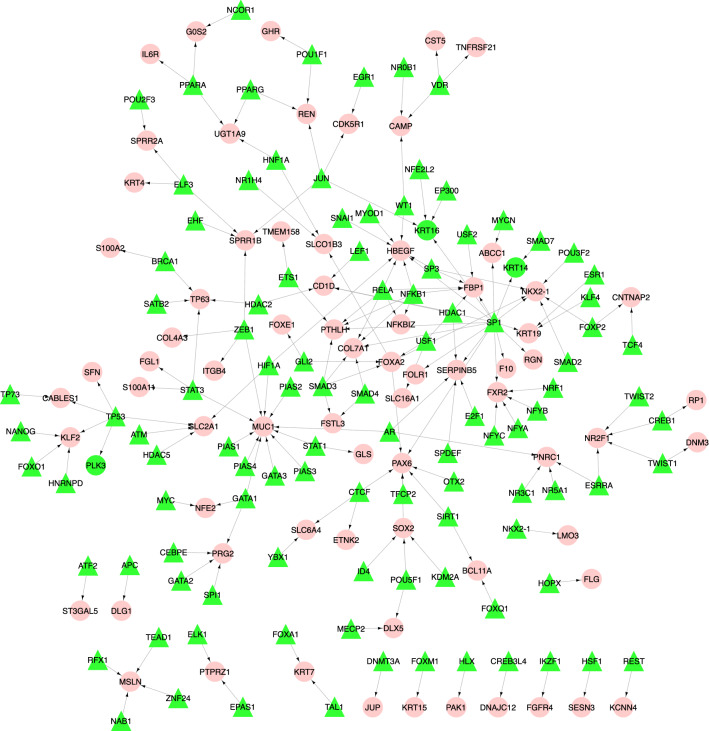
The TF–TG interaction network. This network contains 178 nodes and 182 regulatory interactions. Out of 178 nodes, 107 and 71 nodes are TFs and TGs, respectively. All of the TG nodes are from the *magenta* and *purple* modules. The red circles and green triangles represent TGs and TFs, respectively (this figure was drawn in the Cytoscape^43^ v.3.8.2 software).

In order to identify a list of transcription factor (TF) genes that regulate *magenta* and *purple* modules' genes, the TF–TG regulatory network was reconstructed. Regulatory information of TFs and TGs was retrieved from the TRRUST^40^ online database. After reconstructing the TF–TG regulatory network for *magenta* and *purple* modules, we obtained a network with 178 nodes and 182 regulatory interactions. This network contains 107 TFs and 71 TGs. In this network, *MUC1* with 11 input degrees and *SP1* with 16 output degrees are high TG and TF nodes, respectively. Figure 3 shows the TF–TG regulatory network. A list of TF–TG regulatory interactions is reported in supplementary file S3.

### Drug-TG and Drug-TF Interaction networks

**Figure 4:**
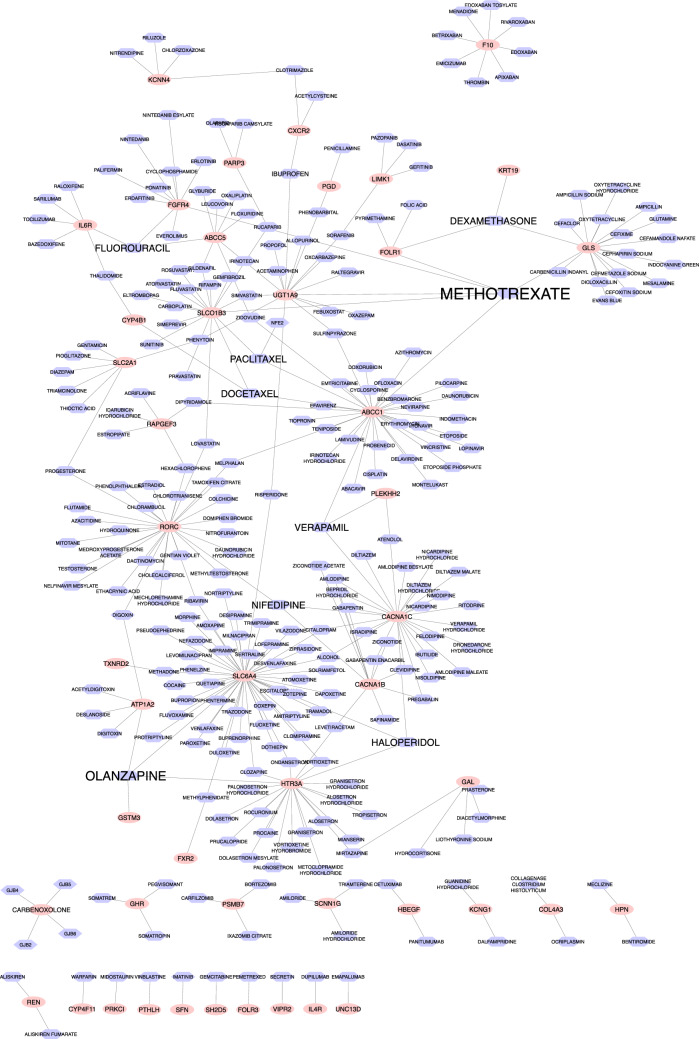
The drug–gene interaction network. Totally, 277 candidate drugs were identified as regulators of the *purple* and *magenta* modules of the NSCLC network. The red circle shapes and blue hexagon shapes represent genes and drugs, respectively (this figure was drawn in the Cytoscape^43^ v.3.8.2 software).

**Figure 5:**
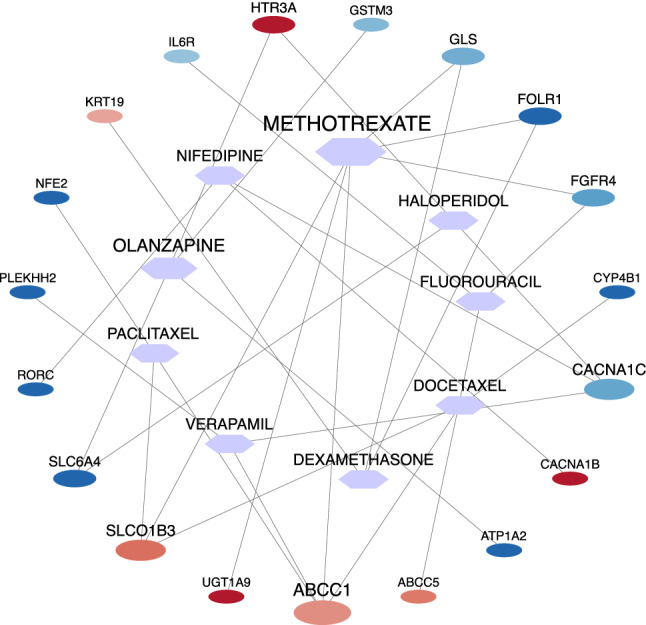
The expression level of hub drugs' target genes in the NSCLC group compared to the normal group. The circle and hexagon shapes represent genes and drugs, respectively. The size of a node indicates its degree (this figure was drawn in the Cytoscape^43^ v.3.8.2 software).

The Drug Gene Interaction Database (DGIdb^42^) was used to detect potential drugs for NSCLC treatment. This database is comprehensive and contains drug–gene interaction information. Using DGIdb, we found 277 candidate drugs that target *purple* and *magenta* modules' genes. These drugs could have a regulatory effect on NSCLC progression. The drug–gene interaction network was reconstructed based on the obtained drugs and the *purple* and *magenta* modules' genes. The Cytoscape^43^ v.3.8.2 software was used to reconstruct and visualize this network. This network is shown in Fig. 4, and further details are reported in Supplementary file S4. This network shows nine drugs, including Methotrexate, Olanzapine, Haloperidol, Fluorouracil, Nifedipine, Paclitaxel *Verapamil*, *Dexamethasone,* and *Docetaxel,* are high-degree nodes. These nine drugs, along with target genes, are selected from the network, and then a sub-network is drawn for these drugs and genes (see Fig. 5). In this sub-network, expression levels of genes in NSCLS samples compared to normal samples are shown with blue (Dow-Regulation) to red (Up-Regulation) colors. Among these genes, *UGT1A9* has the highest up-regulation, and *ATP1A2* has the highest down-regulation expression level in NSCLC group compared to the normal group. METHOTREXATE is a hub node in this sub-network and regulates 6 genes of the *purple* and *magenta* modules. High-degree drugs in the network regulate more genes and can have important regulatory effects. The details of target genes' expression level in 9 drugs of NSCLC group compared to the normal group are reported in Supplementary file S6.

**Figure 6:**
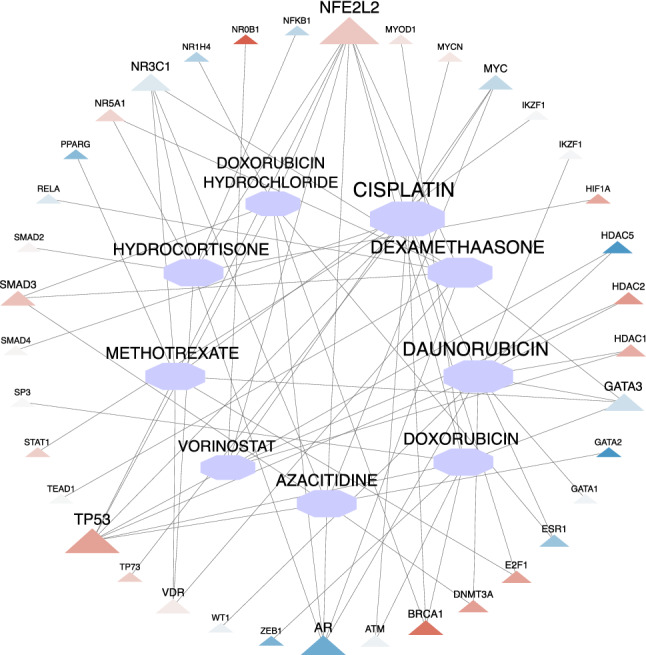
The expression level of hub drugs' target TFs in NSCLC group compared to the normal group. The triangle and hexagon shapes represent TF genes and drugs, respectively. The size of a node indicates its degree (this figure was drawn in the Cytoscape^43^ v.3.8.2 software).

Moreover, a list of drugs that target those TFs regulating *magenta* and *purple* genes was retrieved from the DGIdb database. A list of TFs with regulatory relationships with magenta and purple modules' genes is available in supplementary files S3. Supplementary Fig. D shows the Drug-TF interaction network, and the details of this network are reported in Supplementary file S5. This network contains 723 nodes, including 675 drugs and 48 TFs. The highest degree drug node is *Cisplatin* which regulates 11 TF genes, including *NFE2L2, TP53, ESR1, BRCA1, ATM, MYC, E2F1, SMAD4, MYCN, TP73,* and *STAT1*. *Daunorubicin* is the second highest degree drug node that regulates ten TF genes. Among all drugs, those with degree 7 or above along with target TFs were selected from the network, and then a sub-network was drawn for these drugs and TFs. Figure 6 shows this drug-TF sub-network. In this sub-network, the expression level of TF genes in NSCLC samples compared to normal samples is demonstrated with blue (Dow-Regulation) to red (Up-Regulation) colors. Furthermore, the TFs' expression level in 9 drugs of the NSCLC group compared to the normal group is reported in Supplementary file S6.

In order to investigate and confirm interactions of the candidate drugs and candidate target genes, the DrugBank^45^ database was used. Information for some candidate drugs and candidate target genes is obtained from this database and reported in Table 2. For some other drugs there were no interaction information.

**Table 2:** Confirmation of the candidate drugs and candidate target genes thanks to the DrugBank database.

Drug name	Type	Target gene
Methotrexate	Transporter	Folate receptor alpha (FOLR1)
Methotrexate	Transporter	Solute carrier organic anion transporter family member 1B3 (SLCO1B3)
Olanzapine	Target	5-Hydroxytryptamine receptor 3A (HTR3A)
Paclitaxel	Transporter	Solute carrier organic anion transporter family member 1B3 (SLCO1B3)
Docetaxel	Transporter	Solute carrier organic anion transporter family member 1B3 (SLCO1B3)
Vorinostat	Target	Histone deacetylase 1 (HDAC1)
Vorinostat	Target	Histone deacetylase 1 (HDAC2)

The literature review of the recent articles shows that most of the proposed candidate drugs have significant effects on NSCLC. *Methotrexate and Curcumin are* introduced as novel therapeutic strategies to treat NSCLC^46^. The *Methotrexate* component of MTX-Gd is reported as a chemotherapeutic drug in cancer therapies^47^. Li-Qing Du and colleague noticed that this drug inhibit the expression of RAD51 in cancer cells^48^. Daye Zhang et al. reported that Lenvatinib and Dexamethasone inhibit the invasion and migration of NSCLC^49^. According to Haiyan Ge and colleagues, pemetrexed-induced senescence alleviates in NSCLC by Dexamethasone^50^. In another study, Tatjana Sarcev and colleagues concluded that Dexamethasone significantly decreases weight and appetite in lung cancer patients^51^. Furthermore, Juan P Cata et al. demonstrated that intraoperative Dexamethasone administration to NSCLC patients is not related to its impact on recurrence-free survival (RFS) and overall survival (OS)^52^.

Xin Wang and colleagues revealed that the combination of Ondansetron and Olanzapine has better efficacy in preventing vomiting and chemotherapy-induced nausea in NSCLC patients^53^. According to Thierry André and colleagues, combining Oxaliplatin, Fluorouracil, and Leucovorin could improve colon cancer treatment^54^*.* In a similar study, Herbert Hurwitz et al. reported that Bevacizumab and Fluorouracil composition significantly improved the survival among patients with metastatic colorectal cancer^55^. Furthermore, the combination of Fluorouracil and Curcumin was studied in cancer treatment by Yumeng Wei and colleagues^56^. Barbora Chovancova and colleagues reported that calcium channel blocker Nifedipine inhibits immune escape and colorectal cancer progression^57^. Moreover, in several studies, it has been proved that Nifedipine can promote breast cancer^58,59^.

According to Alan Sandler and colleagues, the combination of Paclitaxel, Bevacizumab, and Carboplatin has a significant survival benefit with the risk of increased treatment-related deaths for NSCLC patients^60^. Moreover, Atsuto Mouri and colleagues reported that the combination of Carboplatin and Paclitaxel could be effective and feasible in patients with SCLC, especially those with interstitial lung disease^61^. In another study, Dongjie Ma et al. showed that Paclitaxel increases the sensitivity of lung cancer cells to lobaplatin^62^.

Chundi Zhang and colleagues reported that Verapamil might change the expression level of NW23 and EGFR in lung cancer by post-transcriptional and transcriptional levels, respectively^63^. In addition, S Merry et al. studied the role of Verapamil in overcoming cytotoxic drug resistance in human lung cancer^64^. Zhiyuan Shen and colleagues expressed that circular RNA Foxo3 reduction promotes chemoresistance and prostates cancer progression to Docetaxel^65^. In another study, Hai-Hong Zhou and colleagues recounted that combining Docetaxel and erastin may offer an effective administration for chemo-resistant ovarian cancer patients^66^. Furthermore, Marta Prieto-Vila et al. reported that Quercetin and Docetaxel combination could be a promising therapeutic approach in breast cancer treatment^67^. Juan Valle and colleagues introduced Cisplatin plus Gemcitabine as an effective option for treating advanced biliary cancer^68^. In another study, Deborah K Armstrong and colleagues noted that Cisplatin and Paclitaxel combination improved survival in patients with ovarian cancer^69^. Moreover, Kazumasa Noda et al. reported that Cisplatin plus Irinotecan could effectively treat small-cell lung cancer^70^. According to Ana Catarina Alves and colleagues, Daunorubicin coactions with membranes of cancer cells^71^. Furthermore, Yuanyuan Wang and colleagues found that Daunorubicin can be an effective strategy in NSCLC^71^ treatment. In a study by Jia Guo and colleagues, the Daunorubicin and Tamoxifen combination was reported as an option to eliminate both cancer stem cells and breast cancer cells^72^. Lilia Antonova and colleagues reported that the expression of the breast cancer susceptibility gene BRCA1 was down-regulated by stress hormone Hydrocortisone in mouse cell line^73^. Yuan Hong and colleagues reported that Doxorubicin and Curcumin combination could be a method for Lung cancer therapy^74^. In a similar study, Abolfazl Akbarzadeh et al. reported the combination of Doxorubicin β-elemene co-loaded as a way to treat lung cancer^75^. Moreover, Vanesa Gregorc and colleagues showed that the NGR-hTNF plus Doxorubicin could be a way for SCLC^76^ treatment. According to Yang Yang and colleagues' report, Trichostatin and Azacitidinecan amalgamation decreased tumorigenic of lung cancer cells^77^. Taofeek K Owonikoko and colleagues found that Vorinostat increased Carboplatin and Paclitaxel activity in NSCLC cells^78^. In Sang Eun Park and colleagues' study, Vorinostat and EGFR‑TKI combination was evaluated in NSCLC to reverse EGFR‑TKI resistance^79^. Furthermore, Chun-Hao Pan and colleagues reported that Vorinostat increased the cisplatin-mediated anticancer effects in SCLC cells^80^. Moreover, Doxorubicin Hydrochloride and Haloperidol were tested on different cancer treatments in humans and other organisms^81–87^.

### Gene set enrichment analysis and candidate drugs validation

In order to validate the proposed drugs for NSCLC treatment, the GSEA was performed based on the Enrichr^88^ database. We considered high-degree drug nodes from the drug-TG sub-network, including *Methotrexate*, *Olanzapine*, *Haloperidol*, *Fluorouracil*, *Nifedipin, Paclitaxel*, *Verapamil*, *Dexamethasone*, and *Docetaxel.* In addition*,* high-degree drug nodes from the drug-TF sub-networks, including *Cisplatin*, *Daunorubicin, Dexamethasone, Methotrexate, Hydrocortisone, Doxorubicin, Azacitidine, Vorinostat,* and *Doxorubicin Hydrochloride,* were considered. Out of these 18 drugs, 2 drugs, including *Dexamethasone* and *Methotrexate,* are common between drug-TG and drug-TF sub-networks. Therefore, 16 drugs are assumed as potent candidate drugs for NSCLC treatment, and the CMAP analysis was performed for these drugs.

The results show that *Methotrexate* and *Paclitaxel* downregulate *GLS2* and *NFE2*, respectively, and *Haloperidol* and *Dexamethasone* up-regulate *HTR3A* and *GLS*, respectively. Moreover, the *Azacitidine* up-regulates the *DNMT 3A* TF gene. Moreover, there was no information regarding other drugs. Romero-Benitez and colleagues studied the impact of paclitaxel on NFE2 in vivo, and they revealed that the expression of NFE2 was up-regulated on day 3^89^. In another study, Anna Schuhmacher et al. ^90^ assessed functional and coding variants of the HTR3A subunits in response to haloperidol. Moreover, Takuma Kusabe and colleagues^91^ reported that the expression level of GLS is reduced by treatment dexamethasone.

## Method

### Dataset and preprocessing

In this study, the transcriptomics data with accession number GSE21933 was downloaded from the Gene Expression Omnibus (GEO) database. This data contains 42 male samples, including 21 normal and 21 primary non-small cell lung cancer (NSCLC) samples. The mean and standard deviation of the age for all samples in healthy and NSCLC are about 70 and 7.8, respectively. The annotation file with accession number GPL6254 was used to assign probes to gene IDs.

Hierarchical clustering was done for the normal and NSCLC samples independently to check outlier samples. Results show no outlier samples among the normal and NSCLC samples (see supplementary Fig. A). Therefore, all 42 samples, including normal and NSCLC, are considered for further analysis.

### Gene co-expression network and gene modules

First of all, differentially expressed genes (GEGs) were calculated between the normal and NSCLC groups applying the adjusted p-value and Benjamini & Hochberg's method based on the GEO2R tool. Overall, 4218 genes with adjusted p_value less than 0.01 were considered as the initial gene list (see supplementary file S1). This gene list was used in gene co-expression network reconstruction.

Then, the gene co-expression network from NSCLC expression data was reconstructed through the Weighted Gene Co-expression Network Analysis (WGCNA^35^) package. This package can reconstruct the gene co-expression network in three different ways: "*signed*", "*unsigned*", and "*signed hybrid*". In this project, the type of gene co-expression network is signed hybrid. To adjust the scale-free property of the network, the β (soft thresholding power beta) parameter is applied in this package.

The soft threshold power beta is determined according to the standard scale-free network^92^. This parameter was set to 7 in NSCLC network (see supplementary Fig. B) to gain the scale independency of the network, where the scale-free index ${R}^{2}$ was 0.9. To extract modules for the gene co-expression network, the hierarchical clustering algorithm was applied in WGCNA (see supplementary Fig. C).

### Module preservation analysis

To analyze module preservation, the ${{\varvec{Z}}}_{{\varvec{s}}{\varvec{u}}{\varvec{m}}{\varvec{m}}{\varvec{a}}{\varvec{r}}{\varvec{y}}}$ score was used. The modules with ${{\varvec{Z}}}_{{\varvec{s}}{\varvec{u}}{\varvec{m}}{\varvec{m}}{\varvec{a}}{\varvec{r}}{\varvec{y}}}<2$ is considered as no preservation^93^. The calculation of the ${{\varvec{Z}}}_{{\varvec{s}}{\varvec{u}}{\varvec{m}}{\varvec{m}}{\varvec{a}}{\varvec{r}}{\varvec{y}}}$ is shown in Eq. (1) ^94^. In this equation, ${Z}_{connectivity}$ and ${Z}_{density}$ are the connectivity and density of the subnetwork, respectively^95^. The ${{\varvec{Z}}}_{{\varvec{s}}{\varvec{u}}{\varvec{m}}{\varvec{m}}{\varvec{a}}{\varvec{r}}{\varvec{y}}}$ score in NSCLC network compared to normal data expression is calculated for all extracted modules (see Table 1). It should be noted that the *grey* module shows the genes which are not assigned to other detected modules. Two modules including *purple* and *magenta* (see Fig. 2) have ${Z}_{summary}<2$ and considered as no preservation modules. The gene list of these modules is reported in supplementary file S1. These genes could have crucial rules in NSCLC.

1 $${Z}_{summary}=\frac{{Z}_{connectivity}+{Z}_{density}}{2}$$

### Enrichment analysis

To identify the biological mechanisms of the genes in *purple* and *magenta* modules, we used functional enrichment analysis based on the DAVID^96,97^ (The Database for Annotation, Visualization, and Integrated Discovery) database. Moreover, a pathway enrichment analysis was done for these modules' genes using the Reactome^98^ pathway database.

### TF–TG regulatory relationships

In order to obtain regulatory information of Transcription factor(TF) genes and target genes(TG), the TRRUST^40^ V2.0 online database was utilized. TRRUST is a manually curated database containing transcriptional regulatory information for mice and humans. This version of TRRUST contains 8444 TF–TG regulatory information of 800 human TFs. After obtaining TF–TG regulatory relationships, a TF–TG network, which contained TFs regulating *magenta* and *purple* modules' genes, was reconstructed.

### Drug–gene interaction network

To identify the candidate drugs that target *purple* and *magenta* genes, the DGIdb^42^ (Drug Gene Interaction Database) was used. This database is connected to 22 other related databases. This database brings back the target genes based on 24 related databases. In the current project, to identify drug–gene interactions information, only experimentally validated interactions were considered.

### Gene set enrichment analysis (GSEA)

The gene set enrichment analysis (GSEA) was performed as a validation method to test whether the proposed candidate drugs can counteract the gene expression perturbations caused by NSCLC. To this end, the Connectivity Map (CMAP^99^) analysis was performed using the Enrichr^88^ database. To perform the CMAP analysis, the genes of *purple* and *magenta* modules were submitted to the Enrichr^88^ database to retrieve up-regulated or down-regulated genes in the cells treated with different drugs. Two datasets of CMAP-up and CMAP-down, which contained the genes up-regulated or down-regulated by different drugs, were extracted. We quested for our proposed candidate drugs in CMAP-up and CMAP-down datasets. Totally, 16 drugs were proposed as potent candidate drugs for NSCLC treatment, which was evaluated using the Enrichr database.

Guangda Li and colleagues^100^ identified some hub genes by combining WGCNA, DEG analysis, and functional enrichment analysis in NSCLC. Moreover, in vitro experiments along with the CMAP database were applied to predict and verify small molecule drugs in NSCLC. These researchers reported cephaeline and Emetine with the potential to overcome resistance using CMAP database. In another study, Ying Zheng et al. ^101^ applied the CMAP database to predict the anesthetic drugs that regulate the differential expression of RNA binding proteins in cervical squamous cell carcinoma.

They reported 65 differentially expressed RNA binding proteins in cervical squamous cell carcinoma. Moreover, they obtained four anesthetics containing procaine, tetracaine, benzocaine, and pentoxyverine. Mengnan Zhao and colleagues^102^ have done a study in order to identify a prognostic ferroptosis and iron-metabolism signature for esophageal squamous cell carcinoma and they identified 20 potential compounds using CMAP database.

Moreover, Hang Yang et al.^103^ have done multi-omics-based research and they used CMAP for chemotherapy drug analysis and screening for drugs which reduce the expression of high-risk genes. In other study, Zetian Gong and colleague^104^, explored several potential small molecule drugs using CMAP based on the mRNAs co-expressed with autophagy-related lncRNAs.

## Discussion

This study applied a gene co-expression network analysis to identify potent candidate drugs for NSCLC treatment. To this end, at first, transcriptomics profiles of normal and NSCLC samples were collected, and 4218 genes with a significantly different expression between normal and NSCLC samples were selected for future analysis. Then, a gene co-expression network analysis was reconstructed based on the WGCNA package. Then, two significant gene modules named *purple* and *magenta* were identified. Next, a list of transcription factor genes regulating these two modules' genes was gathered from the TRUST V2.0 online database, and a TF–TG regulatory network was drawn. Subsequently, a list of existing drugs that target TF–TG network genes was collected from the DGIdb V4.0 database, and then two drug–gene interaction networks, including drug-TF and drug-TG, were drawn. In data collection, 675 and 278 drugs were identified for the drug-TF and drug-TG networks, respectively. Consequently, nine high-degree drugs from the drug-TF and drug-TG networks were selected separately and introduced as potent candidate drugs for NSCLC treatment. Eventually, 16 drugs were introduced as potent candidate drugs to treat NSCLC. Out of 16 selected drugs, nine drugs (*Methotrexate, Olanzapine, Haloperidol, Fluorouracil, Nifedipine, Paclitaxel, Verapamil, Dexamethasone, and Docetaxel*) were selected from the drug-TG network, and nine drugs (*Cisplatin, Daunorubicin, Dexamethasone, Methotrexate, Hydrocortisone, Doxorubicin, Azacitidine, Vorinostat and Doxorubicin Hydrochloride)* were selected from the drug-TF sub-network. Out of these 18 hub drugs, *Methotrexate* and *Dexamethasone* are common in drug-TF and drug-TG networks.

In order to evaluate the gene ontology and biological pathways for *purple* and *magenta* modules' genes, the DAVID online tool was used. *Magenta* and *purple* modules were enriched in 72 and 55 Go terms with a p_value < 0.05, respectively. The results showed that *purple* and magenta modules were more significantly enriched in *phospholipid translocation biological process* with a p_value $\approx$ *0.0007* and *skin development biological process* with a p_value < 1.015e−9, respectively. Moreover, five significant biological process terms of purple module are related to lung and respiratory. These five significant terms are: lung epithelial cell differentiation (p_value < 0.001), lung cell differentiation(p_value < 0.001), lung e*pithelium development (p_value* < *0.004), respiratory system development (p_value* < *0.006),* and *lung development (p_value* < *0.01*). Whereas, none of the significant biological process terms of magenta module are related to lung and respiratory. In conclusion, the purple module genes can be important compared to the magenta module in NSCLC studies.

In addition, a pathway enrichment analysis was done for these two modules based on the REACTOME database. The results show that the purple module was significantly enriched in the "regulation of the insulin secretion" pathway. Three genes of the purple module, including CACNA1C, RAPGEF3, and GNAI2, are involved in the *regulation of the insulin secretion* pathway. Talip Zengin et al.^105^ introduced the RAPGEF3 for prognostic risk prediction according to overall survival time for lung adenocarcinoma patients. Xiao Wang and colleagues^106^ have done genome sequencing analysis for lung adenocarcinoma and introduced CACNA1C as a cancer-related gene. Moreover, they reported that this gene was mutated in lung adenocarcinoma tumor tissue. Furthermore, the magenta module was significantly enriched in five biological pathways, including: *"Gap junction assembly",* "*TP53 Regulates Metabolic Genes","Tandem of pore domain in a weak inwardly rectifying K* + *channels (TWIK)", "Tight junction interactions",* and* "Synthesis of 12-eicosatetraenoic acid derivatives".*

The *"Gap junction assembly"* pathway involves four magenta module genes (*GJB2, GJB4, GJB5, GJB6*). Deng Yun Li et al.^107^ and Seon-Sook Han et al. ^108^ reported that *GJB2* expression is aberrantly higher in Lung adenocarcinoma than in control tissue. In a study that Yi-Pei Lin and colleagues^109^ have done, *GJB4* was reported as a novel biomarker for lung cancer. "TP53 Regulates Metabolic Genes" pathway involves five genes of the magenta module containing *GPX2, SESN3, GLS2, SFN, and TP63*. In their research, Kui Liu et al.^110^ revealed that up-regulation of *GPx2* is correlated with worse overall survival for NSCLC patients. Besides, Shuhao Li and colleagues^111^ reported that *SESN3* has high expression in lung cancer patients compared to healthy patients.

Moreover, this gene was reported as an oncogene in esophageal squamous cell carcinoma cells^112^. Rakibul Islam et al.^107,113^ have done a survival analysis, and their results show a worse overall survival value for *SFN*, and Outcomes show that *SFN* may play a crucial role in the development of NSCLC.* "Tandem of pore domain in a weak inwardly rectifying K*+ *channels (TWIK)"* pathway involves two genes of the magenta module, including *KCNK7* and *KCNK1*. Wen Wang and colleagues^114^ constructed a ceRNA network, and they concluded that *KCNK1* is specific to *LINC00467* in Lung adenocarcinoma. *The "Tight junction interactions"* pathway involves three genes of the magenta module containing PRKCI, *CLDN20*, *and PARD6G*. Yongfeng Wu et al. ^115^ demonstrated that mutation of *PRKCI* and some other genes are identified to be correlated with NSCLC metastasis.

Similarly, Fei Yuan and colleagues^116^ reported that *PARD6G* is differentially expressed between Lung adenocarcinoma and lung squamous cell cancer. Finally, the last significant pathway for the magenta module is "Synthesis of 12-eicosatetraenoic acid derivatives". This pathway contains two genes of *the magenta* module containing *GPX2 and ALOX12B*. Szymon Zmorzyński et al.^117^ showed that the changes in the activity of *the GPX2* isoform might be associated with other cancers development. In another study, Chao Ma et al.^118^ reported that ALOX12B could predict lung adenocarcinoma accurately.

## Conclusion

In conclusion, we used a gene co-expression network analysis to identify potent candidate drugs for the NSCLC treatment in this study. To this end, at first, a gene co-expression network was reconstructed for the transcriptomics data of the NSCLC patients. Then, two significant gene modules, namely *magenta* and *purple,* were discovered from the constructed co-expression network. After that, a *TF-TG* regulatory network was drawn for *magenta* and *purple* modules' genes and the TFs targeting these modules' genes. Next, two drug–gene interaction networks, namely *drug-TG* and *drug-TF,* were constructed. Subsequently, from each *drug-TG* and *drug-TF* network, nine high-degree drugs were selected and reported as potent candidates for NSCLC treatment. Consequently, 16 drugs, including *Methotrexate, Olanzapine, Haloperidol, Fluorouracil, Nifedipine, Paclitaxel, Verapamil, Dexamethasone, Docetaxel*, *Cisplatin, Daunorubicin, Hydrocortisone, Doxorubicin, Azacitidine, Vorinostat,* and *Doxorubicin Hydrochloride,* were introduced as potent candidate drugs to treat NSCLC. Moreover, gene ontology and pathway enrichment analyses were run for the *magenta* and *purple* modules.

## Supplementary Information

Supplementary Information 1.

Supplementary Information 2.

Supplementary Information 3.

Supplementary Information 4.

Supplementary Information 5.

Supplementary Information 6.

Supplementary Figures.

## Data Availability

The corresponding author can provide the datasets utilized in this study on a reasonable request. The raw dataset is available on Information Gene expression Omnibus (GEO) with GSE21933 accession number (https://www.ncbi.nlm.nih.gov/geo/query/acc.cgi?acc=GSE21933).

## References

[1] Nasim F, Sabath BF, Eapen GA (2019). Lung cancer. Med. Clin. N. Am..

[2] Chen Z, Fillmore CM, Hammerman PS, Kim CF, Wong K-K (2014). Non-small-cell lung cancers: A heterogeneous set of diseases. Nat. Rev. Cancer.

[3] Langhammer S (2013). Rationale for the design of an oncology trial using a generic targeted therapy multi-drug regimen for NSCLC patients without treatment options. Oncol. Rep..

[4] Gao X (2019). Estrogen receptors promote NSCLC progression by modulating the membrane receptor signaling network: A systems biology perspective. J. Transl. Med..

[5] Zhao M, Li X, Chen X (2021). GOLM1 predicts poor prognosis of patients with NSCLC and is associated with the proliferation and chemo-sensitivity of cisplatin in NSCLC cells: Bioinformatics analysis and laboratory validation. J. Bioenerg. Biomembr..

[6] Islam R (2021). Identification of molecular biomarkers and pathways of NSCLC: Insights from a systems biomedicine perspective. J. Genet. Eng. Biotechnol..

[7] Zhang Y-Q (2021). Evaluation of the roles and regulatory mechanisms of PD-1 target molecules in NSCLC progression. Ann. Transl. Med..

[8] Begley CG (2021). Drug repurposing: Misconceptions, challenges, and opportunities for academic researchers. Sci. Transl. Med..

[9] Adhami M, Sadeghi B, Rezapour A, Haghdoost AA, MotieGhader H (2021). Repurposing novel therapeutic candidate drugs for coronavirus disease-19 based on protein–protein interaction network analysis. BMC Biotechnol..

[10] MotieGhader H, Safavi E, Rezapour A, Amoodizaj FF (2021). Drug repurposing for coronavirus (SARS-CoV-2) based on gene co-expression network analysis. Sci. Rep..

[11] Soleimani Zakeri NS, Pashazadeh S, MotieGhader H (2021). Drug repurposing for Alzheimer's disease based on protein–protein interaction network. Biomed. Res. Int..

[12] Masoudi-Sobhanzadeh Y, Omidi Y, Amanlou M, Masoudi-Nejad A (2019). DrugR+: A comprehensive relational database for drug repurposing, combination therapy, and replacement therapy. Comput. Biol. Med..

[13] Pushpakom S (2019). Drug repurposing: Progress, challenges and recommendations. Nat. Rev. Drug Discov..

[14] Hooshmand SA (2021). A multimodal deep learning-based drug repurposing approach for treatment of COVID-19. Mol. Divers..

[15] Xue H, Li J, Xie H, Wang Y (2018). Review of drug repositioning approaches and resources. Int. J. Biol. Sci..

[16] Soleimani Zakeri NS, Pashazadeh S, MotieGhader H (2021). Drug repurposing for Alzheimer’s disease based on protein-protein interaction network. BioMed Res. Int..

[17] Ghasemi M, Seidkhani H, Tamimi F, Rahgozar M, Masoudi-Nejad A (2014). Centrality measures in biological networks. Curr. Bioinform..

[18] Moti Ghader, H., KeyKhosravi, D. & HosseinAliPour, A. *Asian Conference on Intelligent Information and Database Systems.* 247–257 (Springer, 2021).

[19] Conte F (2020). A paradigm shift in medicine: A comprehensive review of network-based approaches. Biochim. Biophys. BBA Acta Gene Regulat. Mech..

[20] Kouhsar M, AzimzadehJamalkandi S, Moeini A, Masoudi-Nejad A (2019). Detection of novel biomarkers for early detection of non-muscle-invasive bladder cancer using competing endogenous RNA network analysis. Sci. Rep..

[21] Fiscon G, Conte F, Farina L, Paci P (2021). SAveRUNNER: A network-based algorithm for drug repurposing and its application to COVID-19. PLoS Comput. Biol..

[22] Fiscon G, Paci P (2021). SAveRUNNER: An R-based tool for drug repurposing. BMC Bioinform..

[23] Li X, Li B, Ran P, Wang L (2018). Identification of ceRNA network based on a RNA-seq shows prognostic lncRNA biomarkers in human lung adenocarcinoma. Oncol. Lett..

[24] Peyvandipour A, Saberian N, Shafi A, Donato M, Draghici S (2018). A novel computational approach for drug repurposing using systems biology. Bioinformatics.

[25] Guo W-F (2021). Network controllability-based algorithm to target personalized driver genes for discovering combinatorial drugs of individual patients. Nucleic Acids Res..

[26] Li A, Huang H-T, Huang H-C, Juan H-F (2021). LncTx: A network-based method to repurpose drugs acting on the survival-related lncRNAs in lung cancer. Comput. Struct. Biotechnol. J..

[27] Abedi Z, MotieGhader H, Hosseini SS, Sheikh BeigGoharrizi MA, Masoudi-Nejad A (2022). mRNA–miRNA bipartite networks reconstruction in different tissues of bladder cancer based on gene co-expression network analysis. Sci. Rep..

[28] Jain AS (2021). Everything old is new again: Drug repurposing approach for non-small cell lung cancer targeting MAPK signaling pathway. Front. Oncol..

[29] Thirunavukkarasu MK, Karuppasamy R (2021). Drug repurposing combined with MM/PBSA based validation strategies towards MEK inhibitors screening. J. Biomol. Struct. Dyn..

[30] Boulos JC (2021). Repurposing of the ALK inhibitor crizotinib for acute leukemia and multiple myeloma cells. Pharmaceuticals.

[31] Motieghader H, Kouhsar M, Najafi A, Sadeghi B, Masoudi-Nejad A (2017). mRNA–miRNA bipartite network reconstruction to predict prognostic module biomarkers in colorectal cancer stage differentiation. Mol. BioSyst..

[32] Ahmadi H (2013). HomoTarget: A new algorithm for prediction of microRNA targets in *Homo sapiens*. Genomics.

[33] Li X-T (2021). Gene co-expression modules integrated with immunoscore predicts survival of non-small cell lung cancer. Cancer Treat. Res. Commun..

[34] Wang G (2021). Study of the co-expression gene modules of non-small cell lung cancer metastases. Cancer Biomark..

[35] Langfelder P, Horvath S (2008). WGCNA: An R package for weighted correlation network analysis. BMC Bioinform..

[36] Langfelder P, Horvath S (2008). WGCNA: An R package for weighted correlation network analysis. BMC Bioinform..

[37] Ren W (2021). RYR2 mutation in non-small cell lung cancer prolongs survival via down-regulation of DKK1 and up-regulation of GS1-115G20. 1: A weighted gene Co-expression network analysis and risk prognostic models. IET Syst. Biol..

[38] Chen B, Xie X, Lan F, Liu W (2021). Identification of prognostic markers by weighted gene co-expression network analysis in non-small cell lung cancer. Bioengineered.

[39] Ling B (2020). Identification of prognostic markers of lung cancer through bioinformatics analysis and in vitro experiments. Int. J. Oncol..

[40] Han H (2018). TRRUST v2: An expanded reference database of human and mouse transcriptional regulatory interactions. Nucleic Acids Res..

[41] Huang DW, Sherman BT, Lempicki RA (2009). Systematic and integrative analysis of large gene lists using DAVID bioinformatics resources. Nat. Protoc..

[42] Freshour SL (2021). Integration of the drug–gene interaction database (DGIdb 4.0) with open crowdsource efforts. Nucleic Acids Res..

[43] Shannon P (2003). Cytoscape: A software environment for integrated models of biomolecular interaction networks. Genome Res..

[44] Jassal B (2020). The reactome pathway knowledgebase. Nucleic Acids Res..

[45] Wishart DS (2006). DrugBank: A comprehensive resource for in silico drug discovery and exploration. Nucleic Acids Res..

[46] Rudnik LAC (2020). Co-loaded curcumin and methotrexate nanocapsules enhance cytotoxicity against non-small-cell lung cancer cells. Molecules.

[47] Xu D (2018). Evaluation of methotrexate-conjugated gadolinium (III) for cancer diagnosis and treatment. Drug Des. Dev. Ther..

[48] Du L-Q (2012). Methotrexate-mediated inhibition of RAD51 expression and homologous recombination in cancer cells. J. Cancer Res. Clin. Oncol..

[49] Zhang D, Zhang Y, Cai Z, Tu Y, Hu Z (2020). Dexamethasone and lenvatinib inhibit migration and invasion of non-small cell lung cancer by regulating EKR/AKT and VEGF signal pathways. Exp. Ther. Med..

[50] Ge H (2018). Dexamethasone alleviates pemetrexed-induced senescence in non-small-cell lung cancer. Food Chem. Toxicol..

[51] Šarčev T, Sečen N, Sabo A, Považan Đ (2008). Influence of dexamethasone on appetite and body weight in lung cancer patients. Med. Pregl..

[52] Cata JP (2016). Lack of association between dexamethasone and long-term survival after non-small cell lung cancer surgery. J. Cardiothorac. Vasc. Anesth..

[53] Wang X, Wang L, Wang H, Zhang H (2015). Effectiveness of olanzapine combined with ondansetron in prevention of chemotherapy-induced nausea and vomiting of non-small cell lung cancer. Cell Biochem. Biophys..

[54] André T (2004). Oxaliplatin, fluorouracil, and leucovorin as adjuvant treatment for colon cancer. N. Engl. J. Med..

[55] Hurwitz H (2004). Bevacizumab plus irinotecan, fluorouracil, and leucovorin for metastatic colorectal cancer. N. Engl. J. Med..

[56] Wei Y, Yang P, Cao S, Zhao L (2018). The combination of curcumin and 5-fluorouracil in cancer therapy. Arch. Pharmacal Res..

[57] Chovancova B (2020). Calcium signaling affects migration and proliferation differently in individual cancer cells due to nifedipine treatment. Biochem. Pharmacol..

[58] Zhao T, Guo D, Gu Y, Ling Y (2017). Nifedipine stimulates proliferation and migration of different breast cancer cells by distinct pathways. Mol. Med. Rep..

[59] Guo D-Q, Zhang H, Tan S-J, Gu Y-C (2014). Nifedipine promotes the proliferation and migration of breast cancer cells. PLoS ONE.

[60] Sandler A (2006). Paclitaxel–carboplatin alone or with bevacizumab for non–small-cell lung cancer. N. Engl. J. Med..

[61] Mouri A (2019). Combination therapy with carboplatin and paclitaxel for small cell lung cancer. Respir. Investig..

[62] Ma D (2018). Paclitaxel increases the sensitivity of lung cancer cells to lobaplatin via PI3K/Akt pathway. Oncol. Lett..

[63] Zhang C (2009). Effect of verapamil on the expression of EGFR and NM23 in A549 human lung cancer cells. Anticancer Res..

[64] Merry S, Courtney E, Fetherston C, Kaye S, Freshney R (1987). Circumvention of drug resistance in human non-small cell lung cancer in vitro by verapamil. Br. J. Cancer.

[65] Shen Z, Zhou L, Zhang C, Xu J (2020). Reduction of circular RNA Foxo3 promotes prostate cancer progression and chemoresistance to docetaxel. Cancer Lett..

[66] Zhou H-H (2019). Erastin reverses ABCB1-mediated docetaxel resistance in ovarian cancer. Front. Oncol..

[67] Prieto-Vila M (2020). Quercetin inhibits Lef1 and resensitizes docetaxel-resistant breast cancer cells. Molecules.

[68] Lin, J. *et al.* (American Society of Clinical Oncology, 2020).

[69] Armstrong DK (2006). Intraperitoneal cisplatin and paclitaxel in ovarian cancer. N. Engl. J. Med..

[70] Noda K (2002). Irinotecan plus cisplatin compared with etoposide plus cisplatin for extensive small-cell lung cancer. N. Engl. J. Med..

[71] Alves AC (2017). The daunorubicin interplay with mimetic model membranes of cancer cells: A biophysical interpretation. Biochim. Biophys. Acta Biomembr..

[72] Guo J, Lu W-L (2010). Effects of stealth liposomal daunorubicin plus tamoxifen on the breast cancer and cancer stem cells. J. Pharm. Pharm. Sci..

[73] Antonova L, Mueller CR (2008). Hydrocortisone down-regulates the tumor suppressor gene BRCA1 in mammary cells: A possible molecular link between stress and breast cancer. Genes Chromosom. Cancer.

[74] Hong Y (2019). Lung cancer therapy using doxorubicin and curcumin combination: Targeted prodrug based, pH sensitive nanomedicine. Biomed. Pharmacother..

[75] Cao C, Wang Q, Liu Y (2019). Lung cancer combination therapy: Doxorubicin and β-elemene co-loaded, pH-sensitive nanostructured lipid carriers. Drug Des. Dev. Ther..

[76] Gregorc V (2018). NGR-hTNF and doxorubicin as second-line treatment of patients with small cell lung cancer. Oncologist.

[77] Yang Y, Yin W, Wu F, Fan J (2017). Combination of azacitidine and trichostatin A decreased the tumorigenic potential of lung cancer cells. Onco. Targets. Ther..

[78] Owonikoko TK (2010). Vorinostat increases carboplatin and paclitaxel activity in non-small cell lung cancer cells. Int. J. Cancer.

[79] Park SE (2019). Vorinostat enhances gefitinib-induced cell death through reactive oxygen species-dependent cleavage of HSP90 and its clients in non-small cell lung cancer with the EGFR mutation. Oncol. Rep..

[80] Pan C-H (2016). Vorinostat enhances the cisplatin-mediated anticancer effects in small cell lung cancer cells. BMC Cancer.

[81] Yaşayan G, Mega Tiber P, Orun O, Alarçin E (2020). Doxorubicin hydrochloride loaded nanotextured films as a novel drug delivery platform for ovarian cancer treatment. Pharm. Dev. Technol..

[82] Xiao B (2020). Doxorubicin hydrochloride enhanced antitumour effect of CEA-regulated oncolytic virotherapy in live cancer cells and a mouse model. J. Cell Mol. Med..

[83] Di Francesco M (2021). Doxorubicin hydrochloride-loaded nonionic surfactant vesicles to treat metastatic and non-metastatic breast cancer. ACS Omega.

[84] Friedman GD (2020). Haloperidol and prostate cancer prevention: More epidemiologic research needed. Perm J..

[85] Hui D (2017). Effect of lorazepam with haloperidol vs haloperidol alone on agitated delirium in patients with advanced cancer receiving palliative care: A randomized clinical trial. JAMA.

[86] Radha Krishna LK, Poulose VJ, Goh C (2012). The use of midazolam and haloperidol in cancer patients at the end of life. Singap. Med. J..

[87] Hardy JR (2019). Methotrimeprazine versus haloperidol in palliative care patients with cancer-related nausea: A randomised, double-blind controlled trial. BMJ Open.

[88] Chen EY (2013). Enrichr: Interactive and collaborative HTML5 gene list enrichment analysis tool. BMC Bioinform..

[89] Romero-Benitez MM (2004). In vivo erythroid recovery following paclitaxel injury: Correlation between GATA-1, c-MYB, NF-E2, Epo receptor expressions, and apoptosis. Toxicol. Appl. Pharmacol..

[90] Schuhmacher A (2009). Influence of 5-HT3 receptor subunit genes HTR3A, HTR3B, HTR3C, HTR3D and HTR3E on treatment response to antipsychotics in schizophrenia. Pharmacogenet. Genomics.

[91] Kusabe T (2005). The inhibitory effect of disease-modifying anti-rheumatic drugs and steroids on gliostatin/platelet-derived endothelial cell growth factor production in human fibroblast-like synoviocytes. Rheumatol. Int..

[92] Shi G, Shen Z, Liu Y, Yin W (2020). Identifying biomarkers to predict the progression and prognosis of breast cancer by weighted gene co-expression network analysis. Front. Genet..

[93] Langfelder P, Luo R, Oldham MC, Horvath S (2011). Is my network module preserved and reproducible?. PLoS Comput. Biol..

[94] Riquelme Medina I, Lubovac-Pilav Z (2016). Gene co-expression network analysis for identifying modules and functionally enriched pathways in type 1 diabetes. PLoS ONE.

[95] Pavlopoulos GA (2011). Using graph theory to analyze biological networks. BioData Mining.

[96] Sherman BT, Lempicki RA (2009). Systematic and integrative analysis of large gene lists using DAVID bioinformatics resources. Nat. Protoc..

[97] Huang DW, Sherman BT, Lempicki RA (2009). Bioinformatics enrichment tools: paths toward the comprehensive functional analysis of large gene lists. Nucleic Acids Res..

[98] Fabregat A (2018). The reactome pathway knowledgebase. Nucleic Acids Res..

[99] Lamb J (2006). The Connectivity Map: Using gene-expression signatures to connect small molecules, genes, and disease. Science.

[100] Li G (2021). Identification of hub genes and small molecule drugs associated with acquired resistance to Gefitinib in non-small cell lung cancer. J. Cancer.

[101] Zheng Y, Meng XW, Yang JP (2022). Exploring potential regulatory anesthetic drugs based on RNA binding protein and constructing CESC prognosis model: A study based on TCGA database. Front. Surg..

[102] Zhao M (2022). Identification and analysis of a prognostic ferroptosis and iron-metabolism signature for esophageal squamous cell carcinoma. J. Cancer.

[103] Yang H, Jiang Q (2022). A multi-omics-based investigation of the immunological and prognostic impact of necroptosis-related genes in patients with hepatocellular carcinoma. J. Clin. Lab Anal..

[104] Gong Z, Li Q, Li J, Xie J, Wang W (2022). A novel signature based on autophagy-related lncRNA for prognostic prediction and candidate drugs for lung adenocarcinoma. Transl. Cancer Res..

[105] Zengin T, Önal-Süzek T (2020). Analysis of genomic and transcriptomic variations as prognostic signature for lung adenocarcinoma. BMC Bioinform..

[106] Wang X (2017). Whole genome sequencing analysis of lung adenocarcinoma in Xuanwei, China. Thorac. Cancer.

[107] Li DY, Yue LX, Wang SG, Wang TX (2022). Quercitrin restrains the growth and invasion of lung adenocarcinoma cells by regulating gap junction protein beta 2. Bioengineered.

[108] Han SS (2014). RNA sequencing identifies novel markers of non-small cell lung cancer. Lung Cancer.

[109] Lin YP, Wu JI, Tseng CW, Chen HJ, Wang LH (2019). Gjb4 serves as a novel biomarker for lung cancer and promotes metastasis and chemoresistance via Src activation. Oncogene.

[110] Liu K, Jin M, Xiao L, Liu H, Wei S (2018). Distinct prognostic values of mRNA expression of glutathione peroxidases in non-small cell lung cancer. Cancer Manag. Res..

[111] Li S, Jiang L, Tang J, Gao N, Guo F (2020). Kernel fusion method for detecting cancer subtypes via selecting relevant expression data. Front. Genet..

[112] Li ZW (2020). Small nucleolar RNA host gene 22 (SNHG22) promotes the progression of esophageal squamous cell carcinoma by miR-429/SESN3 axis. Ann. Transl. Med..

[113] Islam R (2021). Identification of molecular biomarkers and pathways of NSCLC: Insights from a systems biomedicine perspective. J. Genet. Eng. Biotechnol..

[114] Wang W, Bo H, Liang Y, Li G (2021). LINC00467 Is Upregulated by DNA Copy Number Amplification and Hypomethylation and Shows ceRNA Potential in Lung Adenocarcinoma. Front. Endocrinol. (Lausanne).

[115] Wu Y (2021). Driver and novel genes correlated with metastasis of non-small cell lung cancer: A comprehensive analysis. Pathol Res Pract.

[116] Yuan F, Lu L, Zou Q (2020). Analysis of gene expression profiles of lung cancer subtypes with machine learning algorithms. Biochim. Biophys. Acta Mol. Basis Dis..

[117] Zmorzyński S, Świderska-Kołacz G, Koczkodaj D, Filip AA (2015). Significance of polymorphisms and expression of enzyme-encoding genes related to glutathione in hematopoietic cancers and solid tumors. Biomed. Res. Int..

[118] Ma C, Li F, Luo H (2021). Prognostic and immune implications of a novel ferroptosis-related ten-gene signature in lung adenocarcinoma. Ann. Transl. Med..

