# Supplementary material for: Drug repositioning in non-small cell lung cancer (NSCLC) using gene co-expression and drug–gene interaction networks analysis
Source: Sci Rep. 2022 Jun 8;12:9417. doi: 10.1038/s41598-022-13719-8 (PMC9177601; doi:10.1038/s41598-022-13719-8)
Supplement: Supplementary file 7 — Supplementary Figures. [file 41598_2022_13719_MOESM7_ESM.docx]

| 1. **Normal**   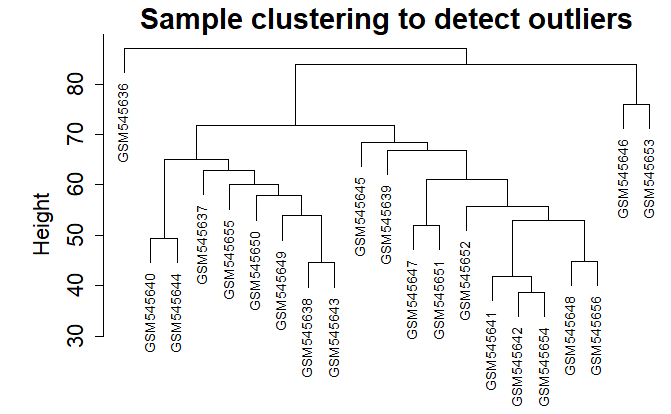 |
| --- |
| 1. **NSCLC**   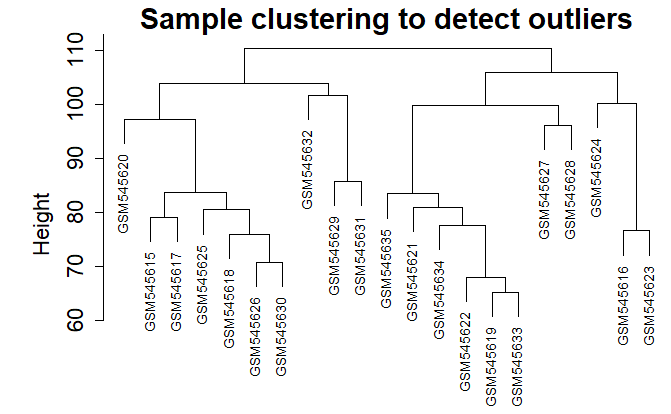 |

**Fig. A** Hierarchical clustering of Normal **(a)** and NSCLC **(b)** samples to detect outliers.

| 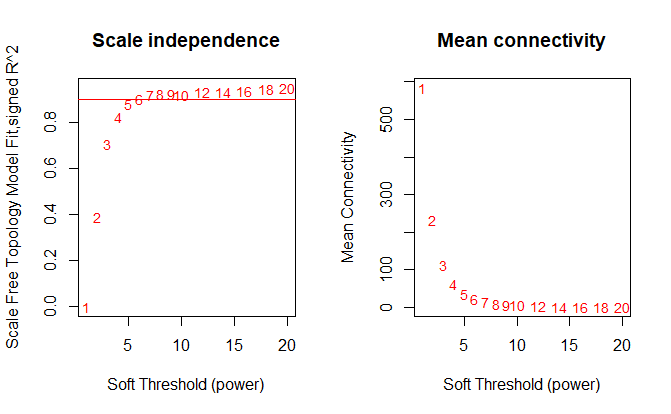 |
| --- |

**Fig. B**  The scale free topology index (left panel) and the mean connectivity (right panel) for different soft-thresholding powers values in NSCLC co-expression networks.

| **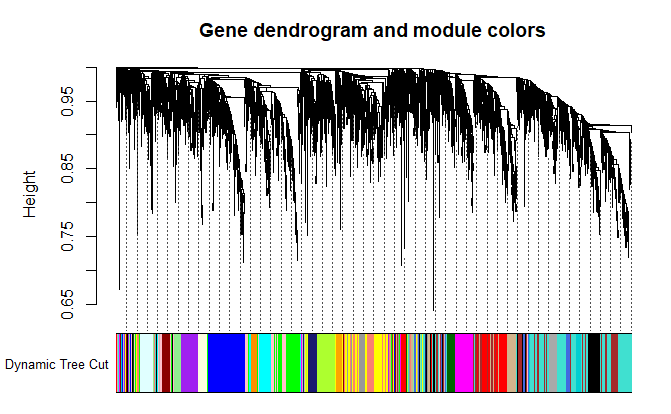** |
| --- |

**Fig. C** Gene co-expression modules in NSCLC. Color band shows predicted modules in the networks**.**


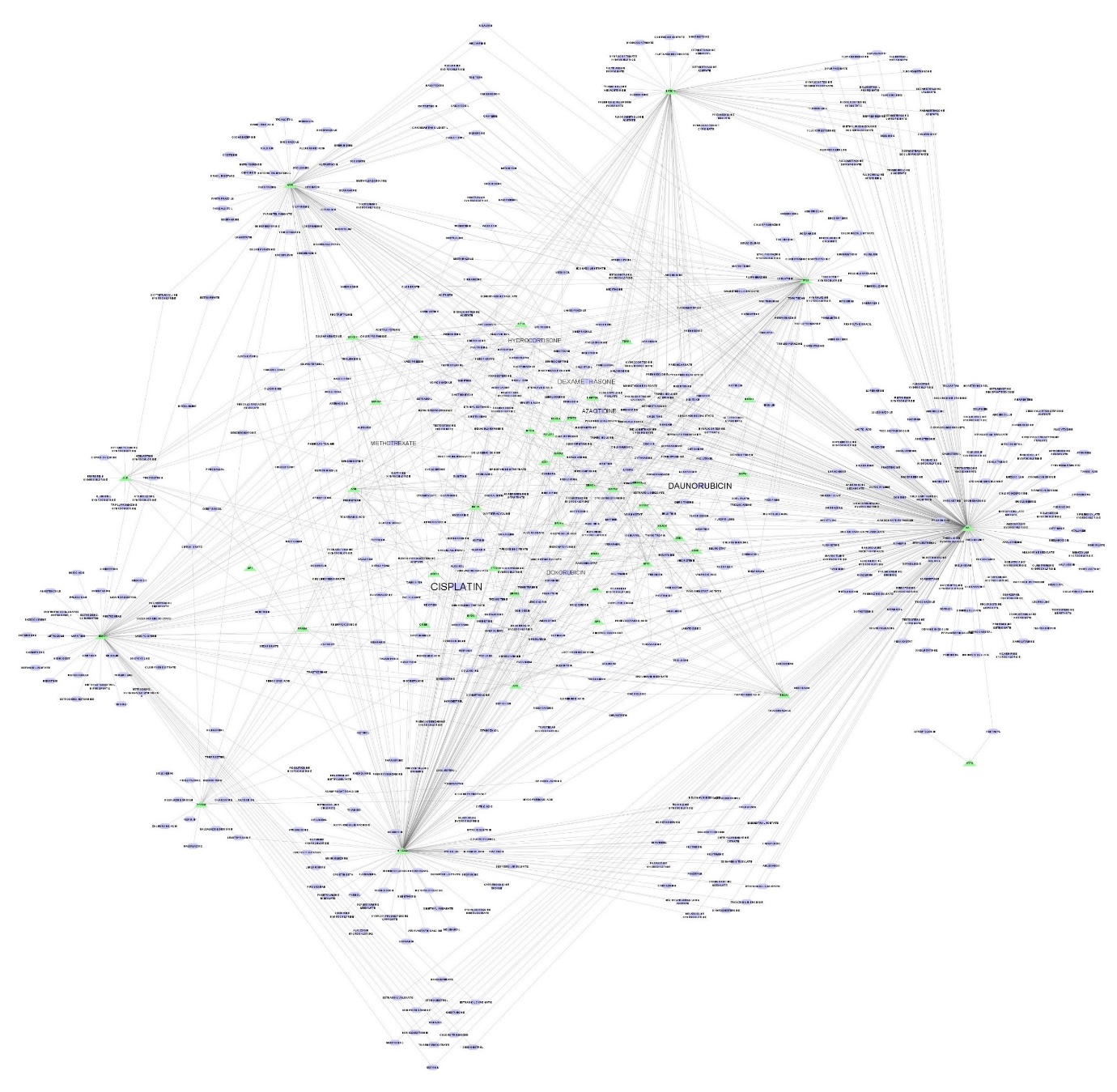


**Fig. D** The Drug-TF interaction network. This network contains 723 nodes with 675 drugs and 48 TFs. High degree nodes of drugs and TFs are *CISPLATIN* and *AR*, respectively. The green triangle shapes and blue hexagon shapes represent TF genes and drugs, respectively.
